# A newly designed intensive caregiver education program reduces cognitive impairment, anxiety, and depression in patients with acute ischemic stroke

**DOI:** 10.1590/1414-431X20198533

**Published:** 2019-09-02

**Authors:** Li Zhang, Tianzhu Zhang, Yan Sun

**Affiliations:** Department of Neurology, The First Affiliated Hospital of Harbin Medical University, Harbin, China

**Keywords:** Acute ischemic stroke, Intensive caregiver education program, Cognitive impairment, Anxiety, Depression

## Abstract

This study aimed to evaluate the effect of a newly designed intensive caregiver education program (ICEP) on reducing cognitive impairment, anxiety, and depression in acute ischemic stroke (AIS) patients. One hundred and ninety-six AIS patients were divided into ICEP group and Control group in a 1:1 ratio using blocked randomization method. In the ICEP group, the caregivers received ICEP, while in the Control group caregivers received usual education and guidance. All patients received conventional rehabilitation treatment. Cognitive impairment (assessed by Mini Mental State Examination (MMSE) score and Montreal Cognitive Assessment (MoCA) score), anxiety (assessed by Hospital Anxiety and Depression Scale (HADS)-A score and Self-rating Anxiety Scale (SAS) score), and depression (assessed by HADS-D score and Self-rating Depression Scale (SDS) score) were assessed at baseline (M0), 3 months (M3), 6 months (M6), and 12 months (M12). Cognitive impairment score at M12 and cognitive impairment score change (M12–M0) were increased, while cognitive impairment rate at M12 was reduced in the ICEP group compared with the Control group. Anxiety score change (M12–M0), anxiety score at M12, and anxiety rate at M12 were decreased in the ICEP group compared with the Control group. Depression score change (M12–M0), depression score at M12, and depression rate at M12 were lower in the ICEP group compared with the Control group. Further subgroup analysis based on baseline features also provided similar results. In conclusion, ICEP effectively reduced cognitive impairment, anxiety, and depression in AIS patients.

## Introduction

Stroke is the leading cause of mortality in the aged population and there are 2.5 million new stroke cases occurring in China annually (1,2). Acute ischemic stroke (AIS) is a major pathological type of stroke triggered by cerebral ischemia, which leads to the dysfunction and degeneration of brain vascular components (3). Currently, the survival rate of AIS has been dramatically improved by some effective AIS therapies (including thrombolysis, endovascular revascularization, AIS reperfusion etc.), while the reduced mortality also increases the number of post-stroke survivors who suffer from complications (2,4). Cognitive impairment, as a complication that affects above one-third of stroke patients, progressively worsens and is accompanied with neuropsychological problems including anxiety and depression (5). In addition, anxiety and depression are also common for post-stroke patients and associated with increased risk of functional dependence as well as reduced quality of life (6). Therefore, effective management to reduce cognitive impairment, depression, and anxiety in AIS patients is essential.

Some evidence indicates that education care programs that are conducted for AIS patients/caregivers are effective in managing AIS complications (7–9). For instance, one education care program accompanied by home-based physical activity, which is performed in post-stroke patients and caregivers, has a positive influence on functional recovery in stroke patients (10). However, the majority of education care programs focus on motor recovery, and there are insufficient studies concerning cognitive and psychological complications, such as cognitive impairment, anxiety, and depression (11). Furthermore, caregivers often experience emotional challenges as well as burden due to the lack of AIS rehabilitation-related knowledge, hence some caregiver education programs are designed to promote the satisfaction of caregivers regarding rehabilitation education (12–15). For example, one caregiver education program promotes caregiving competence as well as working satisfaction in caregivers by establishing caregiving rehabilitation training (16). However, there are limited studies evaluating the efficacy of caregiver education programs on cognitive impairment and psychological disorders in AIS patients (17). In the present study, we designed an intensive caregiver education program (ICEP) that consisted of intensive rehabilitation training, individualized education, as well as psychological nursing, and evaluated the effect of ICEP on cognitive impairment, anxiety, and depression in AIS patients.

## Material and Methods

### Participants

Between January 1, 2014 and December 31, 2016, a total of 196 first-ever AIS patients from The First Affiliated Hospital of Harbin Medical University and their caregivers were consecutively enrolled in this randomized controlled study. The inclusion criteria for AIS patients consisted of: 1) diagnosed as AIS confirmed by brain computed tomography angiography scan, magnetic resonance angiography, or digital-subtraction angiography; 2) able to complete the questionnaire evaluation independently or with the assistance of others; 3) had a fixed family member as nominated caregiver; 4) life expectancy of more than 1 year according to disease severity and re-occurrence risk by clinical experience of clinicians. The exclusion criteria of AIS patients were as follows: 1) secondary AIS; 2) with evidence of hemorrhagic stroke; 3) age younger than 18 years old; 4) with serious cognitive impairment defined as Mini-Mental State Examination (MMSE) score <10; 5) complicated with malignancies or poorly controlled comorbidities. The caregiver was defined as a patient's family member who was willing to participate in the program and most responsible for the patient's daily care. The eligibility of caregivers was determined after consultation with the patients, their family, and the researcher. Caregivers were excluded if they were in poor physical health, had mental or behavioral disorders (such as alcohol abuse, severe orthopedic disability, uncontrolled diabetes, and hypertension), or were unable to understand the study protocol and assist in rehabilitation training. The Institutional Review Board of The First Affiliated Hospital of Harbin Medical University approved the study protocol, and the study was conducted according to ethical standards set forth in the Helsinki Declaration. All participants provided written informed consent before enrollment.

### Randomization

Eligible participants were randomly assigned to the ICEP group (n=98) or the Control group (n=98) in a 1:1 ratio after enrollment using blocked randomization method with a block size of 4. Randomization codes and the participant identification (ID) numbers corresponding to the allocation were created by a statistical analyst from Shanghai Qeejen Bio-tech Company (a medical and statistics service company, China) using SAS 9.4 software (SAS Institute, Inc., USA). The allocation of participants was performed by an independent nurse. On the enrollment of an eligible participant, the nurse got the participant's ID number and allocation information by sending an e-mail to Qeejen for application, then the allocation was revealed, and the appropriate intervention was carried out.

### Intervention for caregivers in the ICEP group

The ICEP was led by trained nurses, assisted by a physical therapist and conducted for a total of 12 months, and the core of ICEP consisted of two sections: 1) intensive individualized education for caregivers and 2) psychological nursing for caregivers. All nurses participating in the ICEP received a one-month training program before the study. After that, there was a skill exam, and only nurses who passed the exam could participate in the current study. Before the initiation of ICEP, basic assessment of the patient's condition was carried out by the physician, which included the assessment of stroke risk factors, complications, consciousness and cognitive function, swallowing function, deep venous thrombosis risk, and emotion. Then, individualized education programs for caregivers were developed by the researchers based on the patient's basic assessment.

For participants in the ICEP group, ICEP was given to the caregiver within 7 days after the patient's hospitalization, which included two stages: hospital stage and discharge stage. In the hospital stage, individualized face-to-face educational sessions were given to the caregiver once a week in the hospital, and each session lasted for one hour. After the patients were discharged from hospital (discharge stage), the caregivers were invited to the hospital every two weeks to receive the individualized educational sessions given by the trained nurse, and each session was 90 min in duration. After each session (both in the hospitalization stage and the discharge stage), there was an additional 30 min psychological nursing for the caregiver, during which the trained nurse would communicate with the caregiver sincerely and attempt to listen, understand, and comfort them, helping them build confidence as well as resolve their troubles and issues. The educational materials were given to the patients and caregivers in the ICEP group after enrollment, and the educational sessions were given according to the materials, which consisted of the following 6 topics: 1) Knowledge of stroke: caregivers were given an introduction of stroke, which included symptoms, causes, risk, impacts, prognosis, therapy, drug use, drug side effects, current management, preventive measures for different kinds of stroke complications, and prevention of secondary stroke; 2) Families' role in caring for patients: caregivers were informed of the potential issues in patients' rehabilitation, and the role as well as the importance of family members and themselves in assisting rehabilitation of the patients were emphasized; 3) Patients' passive emotion management: caregivers were given management techniques regarding stress, unstable mood, anxiety, and depression for patients as well as methods of effective communication; 4) Cognitive rehabilitation training: caregivers were taught methods to help patients perform daily rehabilitation training including training of attention, memory, orientation, calculation, and problem solving; 5) Rehabilitation activities for patients: caregivers were taught skills necessary to assist patients in rehabilitation in three phases: phase 1, help the patient improve joint and muscle conditions, complete muscle strengthening, balance training, and endurance training; phase 2, assist the patient to complete task-specific training of each function with compensatory training methods and encourage the patient to practice the activities in daily living; and phase 3, provide supervision and assistance to prevent falling when the patient takes aerobic exercises; 6) Dietary care: caregivers were taught about healthy diet, healthy cooking methods, identification and management of swallowing difficulties, dysphagia, appropriate method for feeding, feeding through the naso-gastric tube and care of the tube, and ways of increasing food appetite.

### Intervention for caregivers in the Control group

In the Control group, caregivers of patients were given educational materials (as those in the ICEP group) and guidance during hospitalization, which included 1) two face-to-face instruction sessions given by a trained nurse on the day of enrollment and the day of the discharge from hospital; and 2) other appropriate rehabilitation guidance at the request of caregivers. After patients were discharged from hospital, caregivers were followed up by phone calls every three months. At each phone call, the trained nurse inquired about patient's conditions and gave some advice for current rehabilitation.

### Conventional rehabilitation treatment for all patients

All patients who were enrolled in the present study were given conventional rehabilitation treatment based on the basic disease assessment by treating physicians according to the clinical practice guidelines of AIS, which included prevention of stroke reoccurrence and complications, prevention and treatment of spasms, rehabilitation training for motor dysfunction (such as joint range of motion training, good limb position in bed and body position change, traditional muscle strength enhancement training, neurophysiological methods, proprioceptive neuromuscular facilitation, constraint-induced movement therapy, body weight support, treadmill gait training, motor relearning program, etc.), rehabilitation of tactile and proprioceptive disorders, cognitive impairment, emotional disorders, language and communication disorders, dysphagia, urinary and fecal disorders, prevention of deep vein thrombosis, physiotherapy, etc.

### Data collection at baseline

Patients’ characteristics at baseline were recorded after enrollment and included age, gender, education duration, smoking, hypertension, hyperlipidemia, diabetes, as well as lesion location. As for the caregivers, their age, gender, and education duration were collected.

### Outcome measures

To clarify the efficacy of ICEP, patients' cognitive function, anxiety, and depression were assessed by the scales Mini Mental State Exam (MMSE), Montreal Cognitive Assessment (MoCA), Hospital Anxiety and Depression Scale (HADS), Zung Self-rating Anxiety Scale (SAS), and Zung Self-rating Depression Scale (SDS). The assessments of patients' cognitive function, anxiety, and depression were performed at baseline (M0), month 3 (M3), M6, and M12 (if discharged from hospital, the patients were invited to the hospital to complete assessments at those time points) and all scales were completed by patients independently or with the assistance of caregivers. Then, the scores for MMSE, MoCA, HADS-anxiety (HADS-A), HADS-depression (HADS-D), SAS, and SDS were calculated by nurses. For the MMSE and MoCA, a lower score was associated with a more serious cognitive impairment. A cut-off value of ≤26 on MMSE or MoCA was considered indicative of cognitive impairment (18), 21–25 on MoCA was considered mild cognitive impairment (MCI), ≤20 on MoCA was considered indicative of dementia (19), 19–25 on MMSE was indicative of MCI, and 10–18 on MMSE was considered as indicative of moderate cognitive impairment (20). For the HADS-A and HADS-D, a higher score indicated a more severe anxiety or depression, which were classified as 0–7, no anxiety/depression; 8–10, mild anxiety/depression; 11–14, moderate anxiety/depression; 15–21, severe anxiety/depression (21). As for the SAS and SDS, a higher score reflected a severer anxiety or depression, which were classified as: 25–49, no anxiety/depression; 50–59, mild anxiety/depression; 60–69, moderate anxiety/depression; 70–100, severe anxiety/depression (22,23).

### Statistical analysis

The required sample size for this study was estimated based on predictions of a 10% difference between the ICEP group and the Control group in cognitive impairment rate at M12. Assuming a 10% attrition rate and using an alpha level of <0.05, 98 participants per group ensured a power of 80%. All analyses were performed based on the intention-to-treat (ITT) principle with the last observation carried forward (LOCF) method for the missing data. Measurement data are reported as means±SD and compared using the *t*-test between 2 groups; count data are reported as count (percentage) and were compared using the chi-squared test or Wilcoxon rank sum test between 2 groups. Each hypothesis was tested with a 2-tailed analysis and 0.05 as the level of significance. SPSS 21.0 statistical software (SPSS Inc, USA) and GraphPad Prism 6.01 software (GraphPad Software Inc., USA) were used for the statistical data processing and figure making.

## Results

### Study flow

**Figure 1:**
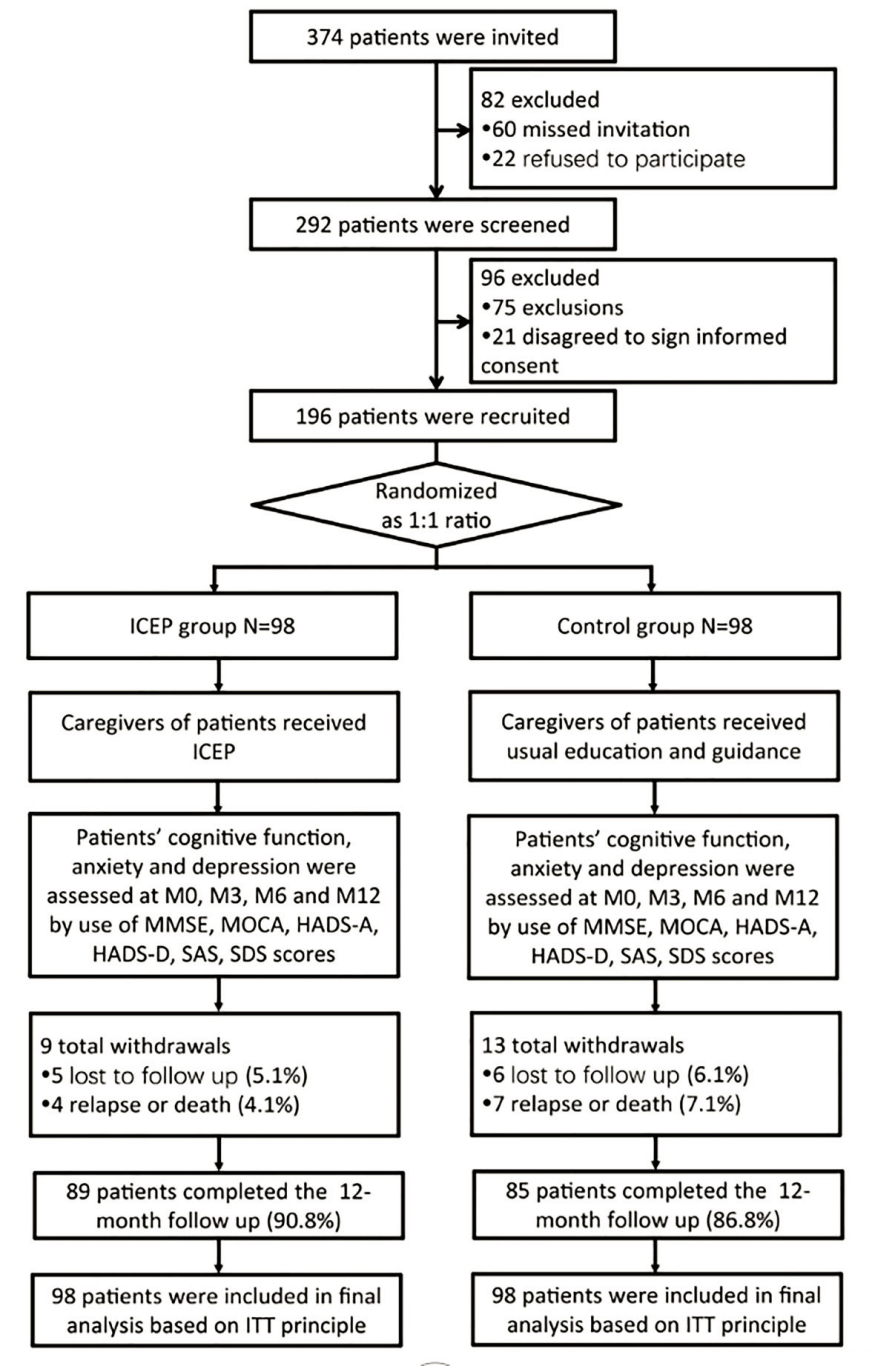
Study flow. ICEP: intensive caregiver education program; M0: baseline; M3: 3 months; M6: 6 months; M12: 12 months; MMSE: Mini Mental State Examination; MoCA: Montreal Cognitive Assessment; HADS-A: Hospital Anxiety and Depression Scale-Anxiety; HADS-D: Hospital Anxiety and Depression Scale-Depression; SAS: Zung Self-rating Anxiety Scale; SDS: Zung Self-rating Depression Scale.

In the current study, 374 AIS patients were invited, while 82 patients were excluded because of refusal to participate (Figure 1). Therefore, 292 patients were screened for eligibility, and 96 patients were excluded of which 75 did not meet inclusion criteria and 21 refused to sign informed consent. Subsequently, the remaining 196 patients were randomized at a ratio of 1:1 into the ICEP group and the Control group. In the ICEP group, 9 patients withdrew, including 5 losses to follow-up (5.1%) and 4 relapses or deaths (4.1%), leaving 89 patients that completed the entire study (90.8%). In the Control group, there were 13 withdrawals, including 6 follow-up losses (6.1%) and 7 relapses or deaths (7.1%), leaving 85 patients who completed the entire study (86.8%). Ultimately, 98 patients in each group were included in final analysis based on the ITT principle.

### Baseline characteristics

There was no difference in the baseline characteristics of caregivers including age, gender, or education level between the ICEP group and the Control group (all P>0.05). The mean ages of caregivers in the ICEP group and the Control group were 47.3±10.4 and 46.6±9.9, respectively (P=0.648). The male/female ratios of caregivers were 30/68 in the ICEP group and 32/66 in the Control group (P=0.759). As to AIS patients, there was no difference in age, gender, lesion location, cognitive impairment, anxiety, or depression between the ICEP group and the Control group (all P>0.05). The detailed baseline characteristics of caregivers and patients of the ICEP and Control groups are listed in Table 1.

**Table 1:** Characteristics of caregivers and patients.

Characteristics	ICEP group (n=98)	Control group (n=98)	P value
Caregivers' characteristics			
Age (years)	47.3±10.4	46.6±9.9	0.648
Gender (male/female)	30/68	32/66	0.759
Highest education (n, %)			0.323
Primary school or less	13 (13.3)	20 (20.4)	
Junior high school	19 (19.4)	23 (23.5)	
Senior high school	23 (23.5)	23 (23.5)	
Undergraduate or above	43 (43.9)	32 (32.7)	
Patients' characteristics			
Age (years)	66.9±9.8	67.3±10.9	0.794
Gender (male/female)	53/45	61/37	0.247
Highest education (n, %)			0.791
Primary school or less	44 (44.9)	47 (48.0)	
Junior high school	24 (24.5)	27 (27.6)	
Senior high school	20 (20.4)	15 (15.3)	
Undergraduate or above	10 (10.2)	9 (9.2)	
Smoking (n, %)	22 (22.4)	23 (23.5)	0.865
Comorbidities (n, %)			
Hypertension	86 (87.8)	79 (80.6)	0.171
Hyperlipidemia	63 (64.3)	60 (61.2)	0.658
Diabetes	19 (19.4)	24 (24.5)	0.388
Lesion location (n, %)			0.583
Left hemisphere	38 (38.8)	39 (39.8)	
Right hemisphere	31 (31.6)	36 (36.7)	
Both hemispheres/brainstem/unknown	29 (29.6)	23 (23.5)	
MMSE score	27.1±2.5	27.0±3.0	0.898
Cognitive impairment by MMSE score (n, %)	26 (26.5)	27 (27.6)	0.872
MoCA score	25.1±3.3	24.5±4.5	0.266
Cognitive impairment by MoCA score (n, %)	54 (55.1)	52 (53.1)	0.774
HADS-A score	6.3±4.1	5.8±4.1	0.428
Anxiety by HADS-A score (n, %)	25 (25.5)	23 (23.5)	0.740
SAS score	42.9±9.7	40.7±10.5	0.139
Anxiety by SAS score (n, %)	22 (22.4)	20 (20.4)	0.728
HADS-D score	6.7±4.5	6.1±4.0	0.311
Depression by HADS-D score (n, %)	30 (30.6)	21 (21.4)	0.143
SDS score	44.2±12.2	43.0±11.6	0.479
Depression by SDS score (n, %)	29 (29.6)	21 (21.4)	0.190

Data are reported as means±SD or count (percentage). Comparisons were determined by the *t*-test or chi-squared test. ICEP: Intensive Caregiver Education Program; MMSE: Mini Mental State Examination; MoCA: Montreal Cognitive Assessment; HADS-A: Hospital Anxiety and Depression Scale-Anxiety; HADS-D: Hospital Anxiety and Depression Scale-Depression; SAS: Zung Self-rating Anxiety Scale; SDS: Zung Self-rating Depression Scale.

### Cognitive impairment in the ICEP group and the Control group

**Figure 2:**
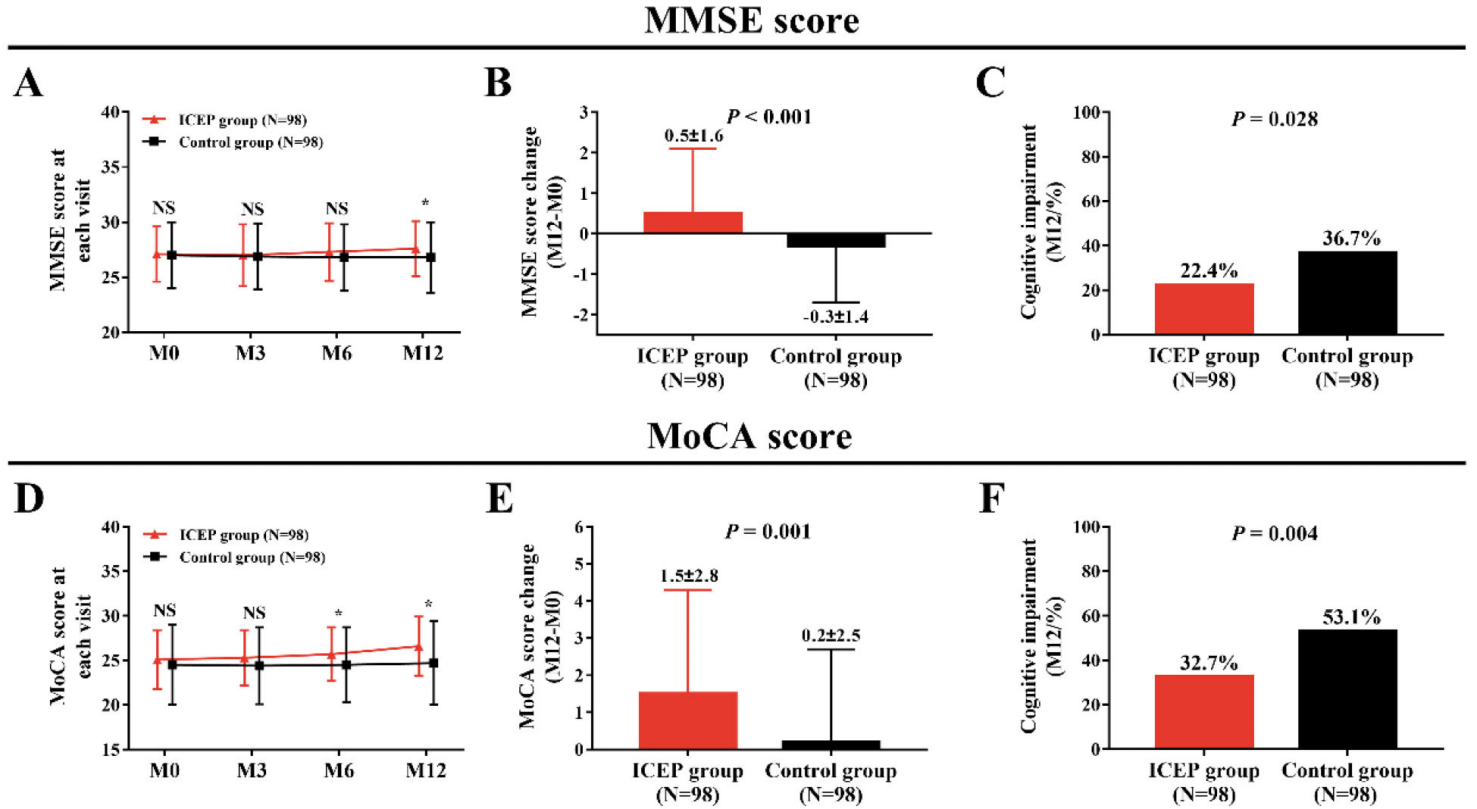
Results of the Mini-Mental State Examination (MMSE) (**A**, **B**, **C**) and Montreal cognitive assessment (MoCA) (**D**, **E**, **F**) in patients with acute ischemic stroke who received the intensive caregiver education program (ICEP) and in Control patients. Data are reported as means±SD. *P<0.05 (*t*-test). NS: non-significant; M0: baseline; M3: 3 months; M6: 6 months; M12: 12 months.

The MMSE score in the ICEP group was similar at M0, M3, and M6 (all P>0.05) but was elevated at M12 (P<0.05) compared with the Control group (Figure 2A). The MMSE score change (M12–M0) was increased in the ICEP group compared with the Control group (P<0.001) (Figure 2B). The cognitive impairment rate at M12 was lower in the ICEP group compared with the Control group (P=0.028) (Figure 2C). The MoCA score in the ICEP group was similar at M0 and M3 (both P>0.05) but was elevated at M6 and M12 (both P<0.05) compared with the Control group (Figure 2D). The MoCA score change (M12–M0) was elevated in the ICEP group compared with the Control group (P=0.001) (Figure 2E), and furthermore, the cognitive impairment rate at M12 was reduced in the ICEP group compared with the Control group (P=0.004) (Figure 2F). Furthermore, since cognitive impairment was strongly influenced by education, comparison of the MMSE score between the ICEP group and the Control group, after adjustment of education level by Propensity Score Matching, was conducted to reduce the influence of education on results, which showed that the ICEP group presented a larger increase in MMSE score (M12–M0) compared with the Control group (P=0.038) (Supplementary Table S1). The above data indicated that ICEP reduced cognitive impairment in AIS patients.

### Anxiety levels in the ICEP group and the Control group

**Figure 3:**
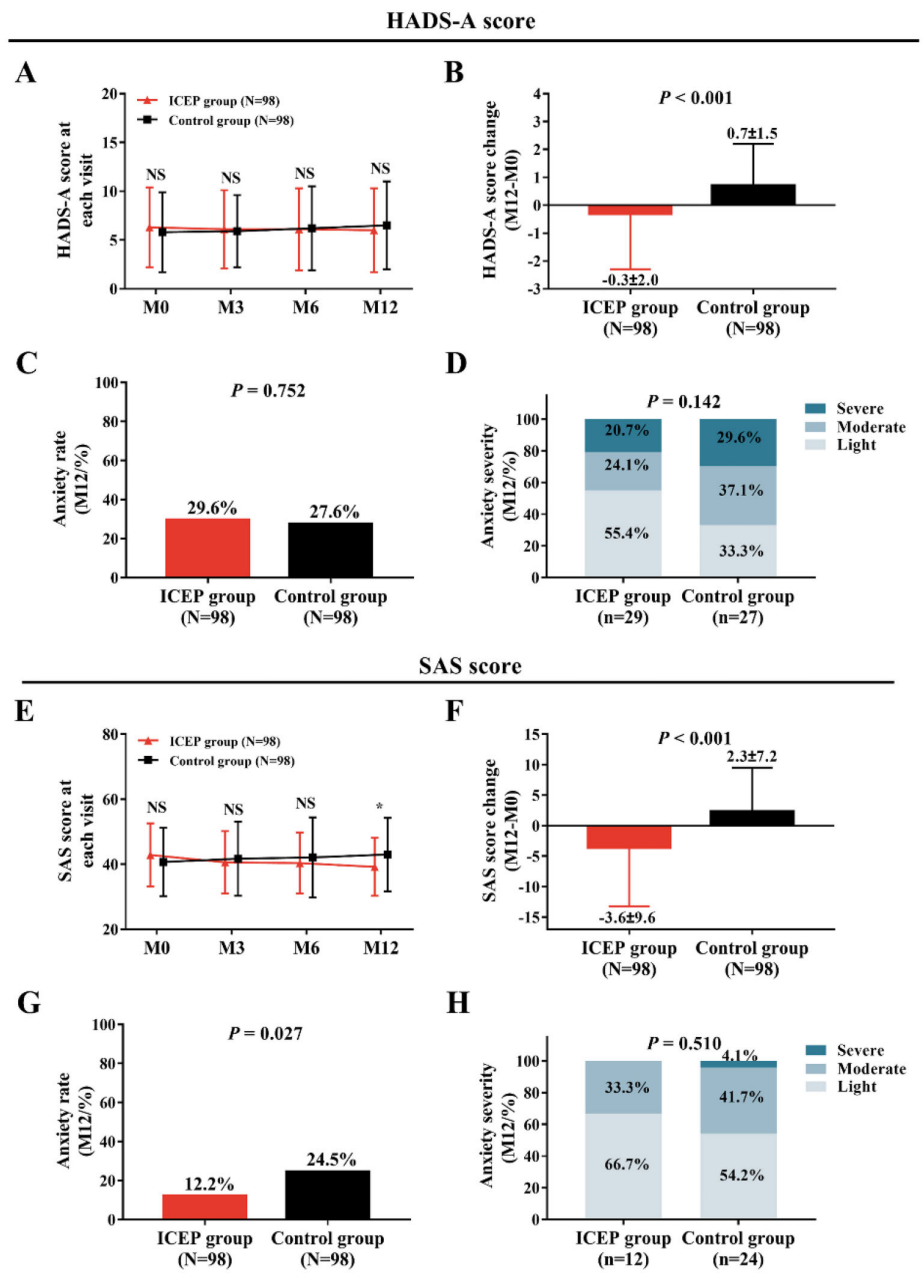
Results of the Hospital Anxiety and Depression Scale-Anxiety (HADS)-A and the Zung Self-rating Anxiety Scale (SAS) in patients with acute ischemic stroke who received the intensive caregiver education program (ICEP) and in Control patients (**A**, **B**, **E**, **F**). Results on anxiety rate and severity are shown in **C**, **D**, **G**, and **H**. Data are reported as means±SD. *P<0.05 (*t*-test). NS, non-significant. M0: baseline; M3: 3 months; M6: 6 months; M12: 12 months.

No difference of HADS-A score between the ICEP group and the Control group was observed at each visit (all P>0.05) (Figure 3A). The HADS-A score change (M12–M0) was decreased in the ICEP group compared with the Control group (P<0.001) (Figure 3B). There was no difference in anxiety rate (P=0.752) (Figure 3C) or anxiety severity at M12 (P=0.142) (Figure 3D) between the ICEP group and the Control group. As for anxiety assessed by the SAS score, the SAS score in the ICEP group had no difference at M0, M3, and M6 (all P>0.05) but was lower at M12 (P<0.05) compared with the Control group (Figure 3E). The SAS score change (M12–M0) was reduced in the ICEP group compared to the Control group (P<0.001) (Figure 3F). Anxiety rate at M12 was decreased in the ICEP group compared with the Control group (P=0.027) (Figure 3G), while there was no difference of anxiety severity at M12 between the ICEP group and the Control group (P=0.510) (Figure 3H). The above data indicated that ICEP decreased anxiety in AIS patients.

### Depression in the ICEP group and the Control group

**Figure 4:**
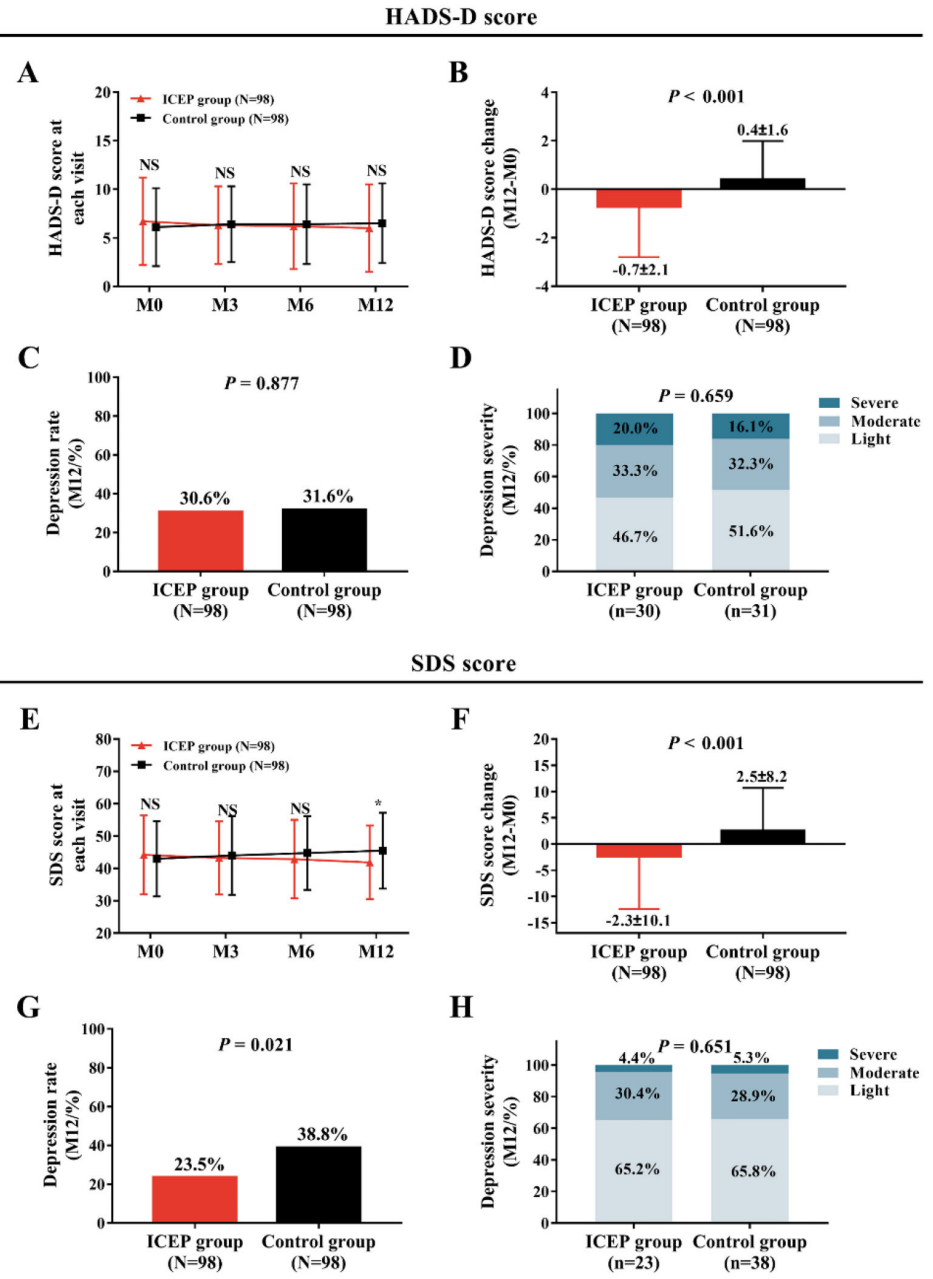
Results of the Hospital Anxiety and Depression Scale-Depression (HADS)-D and the Zung Self-rating Depression Scale (SDS) in patients with acute ischemic stroke who received the intensive caregiver education program (ICEP) and in Control patients (**A**, **B**, **E**, **F**). Results on depression rate and severity are shown in **C**, **D**, **G**, and **H**. Data are reported as means±SD. *P<0.05 (*t*-test). NS: non-significant. M0: baseline; M3: 3 months; M6: 6 months; M12: 12 months.

No difference of HADS-D score was exhibited between the ICEP group and the Control group at each visit (all P>0.05) (Figure 4A). The HADS-D score change (M12–M0) was decreased in the ICEP group compared with the Control group (P<0.001) (Figure 4B). There was no difference in depression rate (Figure 4C) or depression severity (Figure 4D) at M12 between the ICEP group and the Control group (both P>0.05). The SDS score in the ICEP group was similar at M0, M3, and M6 (all P>0.05) but was reduced at M12 (P<0.05) compared with the Control group (Figure 4E). Furthermore, the SDS score change (M12–M0) was decreased in the ICEP group compared with the Control group (P<0.001) (Figure 4F). In addition, depression rate at M12 was reduced in the ICEP group compared with the Control group (P=0.021) (Figure 4G), while depression severity at M12 had no difference between the two groups (P=0.651) (Figure 4H). The above data showed that ICEP reduced depression in AIS patients.

### Longitudinal analyses of ICEP effect on cognitive impairment, anxiety, and depression

To investigate the effect of ICEP in another aspect, we compared the changes of cognitive impairment and anxiety and depression scores from baseline at different time-points between the ICEP group and the Control group. The ICEP group presented a higher reduction in SAS score compared with the Control group at M3, M6, and M12 (all P<0.05) (Supplementary Table S2). The ICEP group exhibited greater reduction in HADS-D score compared with the Control group at M3, M6, and M12 (all P<0.05). However, the ICEP group presented a larger increase in MMSE score (P<0.001) and MoCA score (P=0.001) at M12 compared with the Control group. Detailed information about comparison of cognitive impairment and anxiety and depression score change is presented in Supplementary Table S2. These data indicated that ICEP was effective in reducing anxiety and depression.

### Comparison of cognitive impairment in subgroups

Patients were divided into two subgroups based on MMSE score or MoCA score (patients with and without cognitive impairment at M0) (Table 2). For patients with cognitive impairments at M0, MMSE score change (M12–M0) was higher in the ICEP group compared to the Control group (P=0.002). Cognitive impairment rate at M12 was lower in the ICEP group compared with the Control group (P=0.021). For patients without cognitive impairment at M0, MMSE score change (M12–M0) was elevated in the ICEP group compared with the Control group (P=0.040). For patients with cognitive impairment at M0, MoCA score at M12 and MoCA score change (M12–M0) were increased in the ICEP group compared with the Control group (both P<0.001). Meanwhile, cognitive impairment rate at M12 was lower in the ICEP group compared with the Control group (P<0.001). The above data indicated that ICEP was effective in reducing cognitive impairment in AIS patients with and without cognitive impairment at baseline.

**Table 2:** Subgroup analyses of MMSE and MoCA score in patients with or without cognitive impairment at baseline (M0).

Items	ICEP group	Control group	P value
Based on MMSE score			
Patients with cognitive impairment at M0 (n, %)	26 (26.5%)	27 (27.6%)	
MMSE score (M12)	25.2±3.2	23.3±3.8	0.055
MMSE score change (M12-M0)	1.5±2.2	–0.1±1.3	0.002
Cognitive impairment (M12; n, %)	16 (61.5%)	24 (88.9%)	0.021
Patients without cognitive impairment (M0, n, %)	72 (73.5%)	71 (72.4%)	
MMSE score (M12)	28.5±1.4	28.1±1.5	0.141
MMSE score change (M12-M0)	0.1±1.2	–0.3±1.4	0.040
Cognitive impairment (M12; n, %)	6 (8.3%)	12 (16.9%)	0.122
Based on MoCA score			
Patients with cognitive impairment at M0 (n, %)	54 (55.1%)	52 (53.1%)	
MoCA score (M12)	25.8±3.8	22.1±5.0	<0.001
MoCA score change (M12-M0)	2.9±2.4	1.1±2.7	<0.001
Cognitive impairment (M12; n, %)	20 (37.0%)	42 (80.8%)	<0.001
Patients without cognitive impairment at M0 (n, %)	44 (44.9%)	46 (46.9%)	
MoCA score (M12)	27.5±2.4	27.5±2.0	0.998
MoCA score change (M12-M0)	–0.3±2.2	–0.8±1.9	0.306
Cognitive impairment (M12; n, %)	12 (27.3%)	10 (21.7%)	0.541

Data are reported as means±SD or count (percentage). A P value <0.05 was considered significant (*t*-test or chi-squared test). MMSE: Mini-Mental State Examination; MoCA: Montreal Cognitive Assessment; ICEP: Intensive Caregiver Education Program; M: months.

### Comparison of anxiety in subgroups

Patients were separated into two subgroups based on HADS-A score or SAS score (patients with and without anxiety at M0) (Table 3). For patients with anxiety at M0, HADS-A score change (M12–M0) was reduced in the ICEP group compared with the Control group (P=0.001). For patients with anxiety at M0, SAS score at M12 and anxiety rate at M12 were lower in the ICEP group compared with the Control group (P<0.001, P=0.002), and SAS score change was decreased in the ICEP group compared with the Control group (P<0.001). For patients without anxiety at M0, SAS score change (M12–M0) was decreased in the ICEP group compared with the Control group (P=0.005). The above data showed that ICEP was effective in reducing anxiety in AIS patients with and without anxiety at baseline.

**Table 3:** Subgroup analyses of HADS-A and SAS score in patients with or without anxiety at baseline (M0).

Items	ICEP group	Control group	P value
Based on HADS-A score			
Patients with anxiety at M0 (n, %)	25 (25.5%)	23 (23.5%)	
HADS-A score (M12)	11.0±4.7	13.1±3.7	0.089
HADS-A score change (M12-M0)	–1.1±2.1	1.1±1.9	0.001
Anxiety (M12; n, %)	20 (80.0%)	22 (95.7%)	0.230
Patients without anxiety at M0 (n, %)	73 (74.5%)	75 (76.5%)	
HADS-A score (M12)	4.3±2.5	4.5±2.1	0.748
HADS-A score change (M12-M0)	0.0±1.9	0.5±1.4	0.059
Anxiety (M12; n, %)	9 (12.3%)	5 (6.7%)	0.239
Based on SAS score			
Patients with anxiety at M0 (n, %)	22 (22.4%)	20 (20.4%)	
SAS score (M12)	43.6±11.6	58.8±6.7	<0.001
SAS score change (M12-M0)	–12.8±9.3	1.3±7.7	<0.001
Anxiety (M12; n, %)	7 (31.8%)	16 (80.0%)	0.002
Patients without anxiety at M0 (n, %)	76 (77.6%)	78 (79.6%)	
SAS score (M12)	38.0±7.5	39.0±8.2	0.445
SAS score change (M12-M0)	–1.0±7.9	2.5±7.1	0.005
Anxiety (M12; n, %)	5 (6.6%)	8 (10.3%)	0.412

Data are reported as means±SD or count (percentage). A P value <0.05 was considered significant (*t*-test, chi-squared test, or Wilcoxon rank sum test). HADS-A: Hospital Anxiety and Depression Scale-Anxiety; SAS: Zung Self-rating Anxiety Scale; M: months; ICEP: Intensive Caregiver Education Program.

### Comparison of depression in subgroups

Patients were classified into two subgroups based on HADS-D score or SDS score (patients with and without depression at M0) (Table 4). For patients without depression at M0, HADS-D score at M12 and HADS-D score change (M12–M0) were reduced in the ICEP group compared with the Control group (P=0.012, P=0.001). For patients with depression at M0, SDS score at M12 and SDS score change (M12–M0) were decreased in the ICEP group compared with the Control group (P=0.004, P=0.008) as well as the depression rate at M12 (P=0.013). For patients without depression at M0, SDS score at M12 and score change, as well as depression rate at M12 were reduced in the ICEP group compared with the Control group (P=0.018, P=0.020, P=0.011). The above data showed that ICEP was effective in reducing depression in AIS patients with and without depression at baseline.

**Table 4:** Subgroup analyses of HADS-D and SDS score in patients with or without depression at baseline (M0).

Items	ICEP group	Control group	P value
Based on HADS-D score			
Patients with depression at M0 (n, %)	30 (30.6%)	21 (21.4%)	
HADS-D score (M12)	10.9±3.9	12.3±3.8	0.201
HADS-D score change (M12-M0)	–1.2±2.3	–0.1±1.7	0.090
Depression (M12; n, %)	20 (66.7%)	18 (85.7%)	0.125
Patients without depression at M0 (n, %)	68 (69.4%)	77 (78.6%)	
HADS-D score (M12)	3.9±2.7	4.9±2.4	0.012
HADS-D score change (M12-M0)	-0.5±2.0	0.5±1.6	0.001
Depression (M12; n, %)	10 (14.7%)	13 (16.9%)	0.720
Based on SDS score			
Patients with depression at M0 (n, %)	29 (29.6%)	21 (21.4%)	
SDS score (M12)	51.3±12.1	60.0±7.8	0.004
SDS score change (M12-M0)	–7.3±9.3	–0.5±7.4	0.008
Depression (M12; n, %)	17 (58.6%)	19 (90.5%)	0.013
Patients without depression at M0 (n, %)	69 (70.4%)	77 (78.6%)	
SDS score (M12)	38.0±8.5	41.6±9.3	0.018
SDS score change (M12-M0)	–0.2±9.7	3.3±8.2	0.020
Depression (M12; n, %)	6 (8.7%)	19 (24.7%)	0.011

Data are reported as means±SD or count (percentage). A P value <0.05 was considered significant (*t*-test, chi-squared test, or Wilcoxon rank sum test). HADS-D: Hospital Anxiety and Depression Scale-Depression; SDS: Zung Self-rating Depression Scale; M: months; ICEP: Intensive Caregiver Education Program.

### Depression and anxiety in the ICEP group and the Control group among patients with different cognitive status at baseline

Additionally, patients were divided according to different cognitive status assessed by MoCA and MMSE scores at baseline. According to MoCA score, there were 90 (45.9%) patients without cognitive impairment, 81 (41.3%) with MCI, and 25 (12.7%) with dementia at baseline (Supplementary Table S3). ICEP was effective in reducing anxiety and depression among patients with different cognitive status, especially in patients with MCI. According to MMSE score, there were 143 (72.9%) patients without cognitive impairment, 50 (25.5%) patients with MCI, and 3 (1.5%) patients with moderate cognitive impairment at baseline (Supplementary Table S4). ICEP was effective in reducing anxiety and depression among patients with different cognitive status, especially in patients without cognitive impairment at baseline (Supplementary Tables S3 and S4).

## Discussion

In the present study, all analyses were performed based on the ITT principle with the LOCF method for the missing data, showing that ICEP decreased cognitive impairment (assessed by MMSE score and MoCA score) and reduced anxiety (assessed by HADS-A score and SAS score) and depression (assessed by HADS-D score and SDS score) in AIS patients. Analysis based on per protocol principle also indicated the same trend.

Cognitive impairment is an AIS complication related to reduced functional ability and negative rehabilitation outcome (17). AIS cognitive impairment likely results from cerebral vascular injuries, and may lead to limitation of autonomy as well as dementia in the long-term (24). Therefore, multiple care programs are designed to reduce cognitive impairment in AIS patients. For example, one study presents the positive influence of a customized home-based computerized training on cognitive function in stroke patients including AIS (25). Currently, most rehabilitation programs for cognitive impairment mainly focus on stroke patients instead of caregivers, and the education needs of caregivers often lack attention. Therefore, we speculated that the intensive caregiver education including intensive rehabilitation training, individualized education as well as psychological nursing might have a positive influence on cognitive recovery in AIS patients. In the present study, we found that ICEP reduced the cognitive impairment effectively in AIS patients compared with control, even after adjusting for educational level. The possible reasons might be: 1) the intensive and specialized education about cognitive rehabilitation training for caregivers included in ICEP helped caregivers master necessary cognitive knowledge to guide cognitive training, hence reduced cognitive impairment in AIS patients; and 2) the intensive contact with multidomain experts included in ICEP might improve the confidence of the caregivers and maintain their mental health to help them better perform essential care, thereby enhancing the recovery of cognitive function in AIS patients.

Emerging evidence indicates that psychological disorders including anxiety and depression are prevalent and affect about 20-50% of all AIS patients, which are likely the consequences of vascular lesions or negative emotional reactions to the disease (26–28). Moreover, anxiety and depression are negatively associated with cognitive conditions and neurological outcomes, thus causing psychological burden in AIS patients and their caregivers (29–31). Existing rehabilitation/education programs that include education, counseling, and service from physical therapists or trained nurses are effective in relieving anxiety and depression in AIS patients. For instance, one randomized controlled study elucidates that home rehabilitation program (including a 3-month exercise program and counseling for patients and caregivers) contributes to a higher quality of life and reduced level of depression in patients with ischemic stroke including AIS (32). Although much effort has been done to fill the psychological needs of AIS patients, the needs of caregivers for individualized rehabilitation education and psychological nursing lack attention. We found that anxiety and depression were reduced by ICEP in AIS patients. Interestingly, we found that ICEP was effective in reducing anxiety and depression among patients with different cognitive status. The possible reasons might include: 1) intensive contacts with trained nurses and physical therapists could help caregivers to improve problem-solving ability and learn how to communicate effectively with patients and guide patients to express negative emotions, which relieve the emotional distress and subsequently reduce anxiety and depression in AIS patients; 2) ICEP emphasized the role and importance of family members in comforting and supporting AIS patients, as well as provided psychological nursing to caregivers, which improved their confidence and mental health, thereby reducing the anxiety and depression in AIS patients; and 3) co-occurrence of cognitive impairment and depression.

There were several limitations in this study: 1) This study was single-centered; recruiting patients from multiple centers could validate the results; 2) The follow-up duration of ICEP lasted 1 year, which was relatively short, thus, the long-term benefits of ICEP on cognitive impairment, anxiety, and depression in AIS patients were unclear; 3) This study assessed the cognitive impairment, anxiety, and depression in AIS patients, however, it lacked the assessment of emotional status in caregivers, which might influence AIS patients indirectly; 4) As the AIS patients with serious cognitive impairment were excluded, the result might not be applicable to all AIS patients with cognitive impairments; 5) Since the patients were first-diagnosed AIS patients and most were not previously treated patients in our hospital before enrollment, the record of patients' cognitive impairment before AIS was not accessible, which might be a confounding factor in our study; 6) The caregivers were given psychological nursing by trained nurses rather than by psychologists, which might lead to reduced assessment accuracy compared to psychologists, however, the nurses were well-trained and received the certification of this ICEP program, therefore their professional standards of psychological nursing were valid to a certain extent.

In conclusion, ICEP effectively reduced cognitive impairment, anxiety, and depression in AIS patients. The study indicated that caregivers play important roles during rehabilitation in AIS patients and the AIS education program should take caregivers into consideration.

## Supplementary Material

Click here to view [pdf].
